# Einfluss von künstlicher Intelligenz auf Entscheidungen in der Radiologie am Beispiel des Lungenkrebsscreenings

**DOI:** 10.1007/s00117-025-01496-0

**Published:** 2025-08-01

**Authors:** Marcel Opitz, Luca Salhöfer, Johannes Haubold, Isabel Molwitz, Marco Das

**Affiliations:** 1 Institut für Diagnostische und Interventionelle Radiologie und Neuroradiologie, Universitätsmedizin Essen, Hufelandstraße 55, 45147 Essen, Deutschland; 2 Klinik und Poliklinik für Diagnostische und Interventionelle Radiologie und Nuklearmedizin, Universitätsklinikum Hamburg-Eppendorf, Martinistraße 52, 20246 Hamburg, Deutschland; 3 Klinik für Diagnostische und Interventionelle Radiologie, Helios Klinikum Duisburg GmbH, Dieselstr. 185, 47166 Duisburg, Deutschland

Künstliche Intelligenz (KI) hat das Potenzial, die Patientenversorgung in der modernen Medizin zu revolutionieren. Aufgrund der großen und relativ standardisierten Datenmengen in der Radiologie können Programme auf Basis von KI schon heute im klinischen Alltag Entscheidungsprozesse unterstützen. Aktuell sieht die Lungenkrebs-Früherkennungs-Verordnung (LuKrFrühErkV) für das unmittelbar vor der Einführung stehende Lungenkrebsscreening in Deutschland erstmals die verpflichtende Verwendung einer KI-basierten Unterstützung vor. Hierbei stellen Themenkomplexe wie Nachvollziehbarkeit der KI-Entscheidung, Integration in den radiologischen Arbeitsablauf, Ethik- und Datenschutzaspekte sowie eine wirtschaftliche Abwägung Herausforderungen für die unmittelbare Implementierung dar.

## Einführung eines Programms zur Lungenkrebs-Früherkennung in Risikopopulationen mittels Low-dose-CT

In Deutschland werden jährlich mehr als 1,5 Mio. Computertomographien (CT) des Thorax durchgeführt [1]. Mit der bevorstehenden Einführung eines systematischen Lungenkrebsscreenings ist davon auszugehen, dass die Anzahl der Thorax-CT-Untersuchungen weiter ansteigen wird [2]. Schätzungen zufolge erfüllen in Deutschland circa 5,5 Mio. aktuelle oder ehemalige Raucher die NELSON-Kriterien (50 bis 75 Jahre, aktive oder ehemalige Raucher mit mindestens 15 Packungsjahren) und damit die Voraussetzungen für das Screeningprogramm, wie repräsentative Umfragedaten zum Rauchen zeigen [3]. In dieser Risikogruppe sind knapp die Hälfte aller Lungenkrebsfälle zu erwarten [4].

**Abb. 1:**
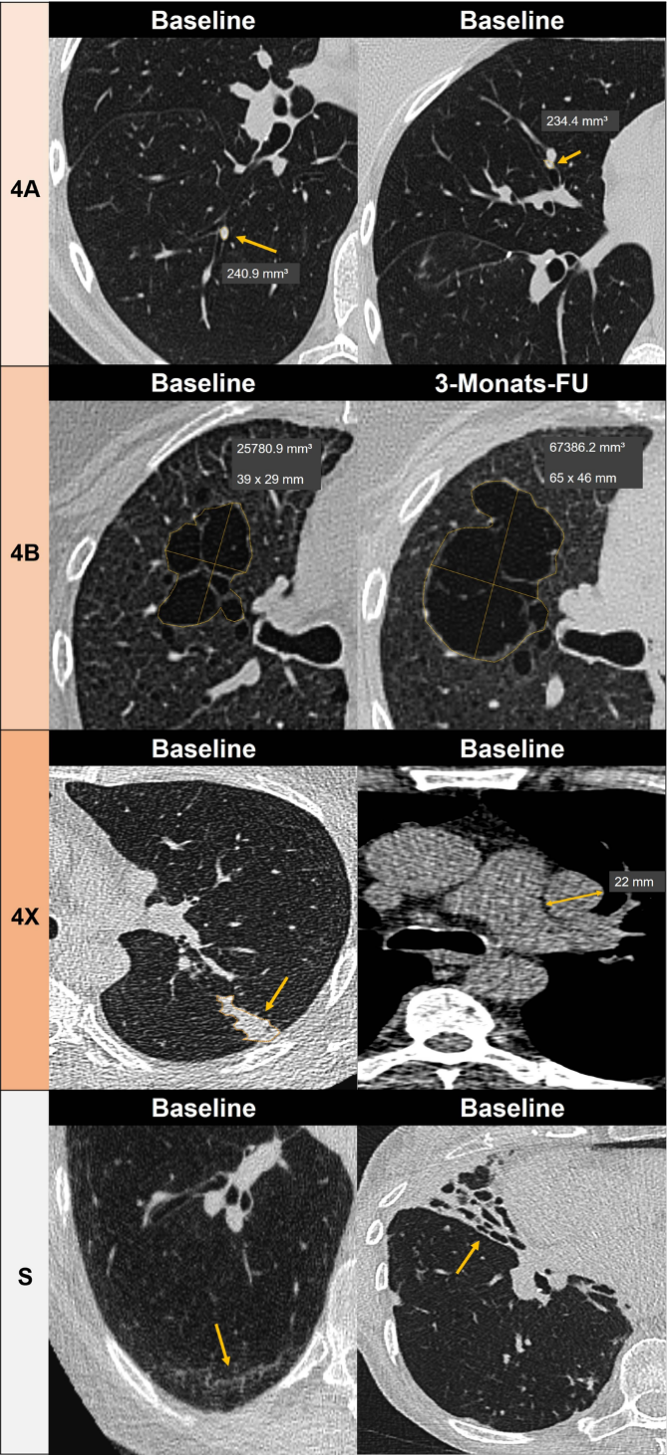
Beispiele für Lungenläsionen unter Anwendung von Lung-CAD-Software mit Klassifikation gemäß Lung-RADS: *4A* Solide endobronchiale Rundherde segmental im rechten Unterlappen (links) und Mittellappen (rechts) in der Baseline-LDCT, histologisch: typisches Karzinoid. *4B* Multilokuläre Zyste mit deutlicher Größenzunahme und vermehrter Septierung in der 3‑Monats-Follow-up-LDCT, histologisch: adenoid-zystisches Lungenkarzinom. *4X* Segmentale Obstruktion eines linken Oberlappenbronchus mit ipsilateraler mediastinaler Lymphadenopathie, histologisch: kleinzellig neuroendokrines Karzinom, Stadium T2aN2M0. *Modifier* *S *(Klinisch relevante Befunde ohne Hinweis auf Lungenkrebs). *Links* Interstitielle Lungenanomalien (ILA), *Rechts* Bronchiektasen

Die Herausforderung im Lungenkrebsscreening liegt nicht nur in der Detektion pulmonaler Rundherde, sondern vor allem in der präzisen Volumetrie und der standardisierten Zuordnung der Befunde gemäß der Lung-RADS-Klassifikation (Abb. 1) sowie in der Verlaufsbeurteilung bei Follow-up-Untersuchungen [5]. Diese Schritte erfordern einen erheblichen zeitlichen Aufwand und sind fehleranfällig, insbesondere bei steigender Anzahl von Untersuchungen.

Der Einsatz von KI in Form einer Lung-CAD-Software (Computer-Aided Detection) bietet eine vielversprechende Lösung. Sie ermöglicht eine automatisierte und standardisierte Analyse von CT-Daten und unterstützt dabei, potenziell bösartige Läsionen schneller und zuverlässiger zu identifizieren. Damit markiert die Einführung von KI-gestützter Technologie im Lungenkrebsscreening einen Meilenstein in der klinischen Translation von KI-Anwendungen und verdeutlicht die wachsende Bedeutung von KI für die medizinische Diagnostik.

## Potenzial und Limitationen einer Lung-CAD-Software für das Lungenkrebsscreening

Aktuelle Studien zum Einsatz von Lung-CAD-Softwares im Lungenkrebsscreening zeigen, dass die Integration solcher Systeme in den radiologischen Workflow das Potenzial bietet, auf eine radiologische Zweitbefundung zu verzichten. Die Kombination aus Radiolog:in und KI-System ermöglichte in prospektiven Screening-Settings eine zuverlässige Befundung, wobei weniger als 5 % der Teilnehmenden nach der initialen Thorax-CT zur weiteren Abklärung an ein spezialisiertes Zentrum überwiesen werden mussten [6]. Daten aus der europäischen Multicenterstudie „4-IN-THE-LUNG-RUN“ belegen zudem, dass eine Lung-CAD-Software das Potenzial hat, Radiolog:innen als Erstleser im Rahmen eines Lungenkrebsscreening-Programms zu ersetzen, indem unauffällige Fälle bereits durch die KI ausgeschlossen werden – ohne das Risiko für falsch-negative Befunde wesentlich zu erhöhen [7].

Gleichzeitig zeigen andere Studien, dass die diagnostische Leistungsfähigkeit solcher KI-Systeme stark von der Erfahrungsebene der Radiolog:innen abhängen kann und nicht in allen Fällen überlegen ist [8]. Der Einsatz von KI-Systemen kann – entgegen häufiger Erwartungen – das Burnout-Risiko unter Radiolog:innen sogar erhöhen, etwa durch gesteigerte Arbeitsdichte und reduzierte Entscheidungsspielräume [9].

Darüber hinaus erlauben einige Lung-CAD-Softwares die simultane Quantifizierung von Koronarkalk und Lungenemphysem und unterstützen so eine umfassende Risikostratifizierung. Beide Parameter gelten in personalisierten Screening-Kohorten als unabhängige Prädiktoren der Gesamtmortalität und erweitern den diagnostischen Nutzen über die reine Lungenkrebsfrüherkennung hinaus [10]. Neben einer LUNG-CAD-Software können KI-basierte Programme das Potenzial des opportunistischen Screenings bzw. der Risikostratifizierung für kardiovaskuläre Ereignisse oder die Gesamtsterblichkeit trotz der niedrigen Dosis der Thorax-CT mittels einer akkuraten Analyse der Körperzusammensetzung erhöhen [11, 12].

Allerdings weisen die derzeit auf dem Markt verfügbaren Lung-CAD-Softwares relevante Limitationen auf. Die zugrundeliegenden KI-Algorithmen sind auf unterschiedlichen Trainingsdatensätzen mit uneinheitlichen Akquisitions- und Rekonstruktionsparametern entwickelt worden. Dies kann sich negativ auf die Interoperabilität und die Vergleichbarkeit der Ergebnisse auswirken. Ergebnisse der HANSE-Studie zeigen deutliche Unterschiede zwischen verschiedenen KI-Systemen in der Detektion, Quantifizierung und Klassifikation pulmonaler Rundherde, was die Notwendigkeit einer sorgfältigen Validierung und Standardisierung der KI-Systeme unterstreicht [13].

## Wirtschaftliche Aspekte in der Nutzung von KI-Tools für die Lungenkrebs-Früherkennung

In Zeiten steigender Energiekosten, drohender Insolvenz zahlreicher Krankenhäuser und vor dem Hintergrund der kürzlich durch den Deutschen Ärztetag verabschiedeten Novellierung der Ärztlichen Gebührenordnung ist Wirtschaftlichkeit imperativ für die Radiologie. KI-Tools für das Lungenkrebsscreening sollten entsprechend nicht nur kostendeckend sein, sondern idealerweise auch einen geringen ökonomischen Nutzen ermöglichen. Die Einflussfaktoren auf die Wirtschaftlichkeit einer solchen Software sind multifaktoriell. Dazu gehören der Kaufpreis, das Kaufmodell (einmaliger Kauf vs. Bezahlung pro Fall; [14, 15]), Installations- und Wartungskosten, Sensitivität und Spezifität des KI-Tools, da diese bei übersehenen Tumoren oder bei unnötiger weiterer Diagnostik Folgekosten bedingen [16], und die Zahlungsbereitschaft der Kostenträger, respektive der Krankenkassen, für potenzielle Zusatzkosten. Während die Überlegenheit einer Volumetrie gegenüber 2D-Messungen von Lungenherden für die Lung-RADS-Klassifikation erwiesen ist und die Zeitersparnis von KI-Tools evident ist, sind diese dennoch nicht inhärent kosteneffektiv. So können beispielsweise die Softwarekosten den ökonomischen Mehrwert einer Zeitersparnis aufheben. Während für einzelne KI-Anwendungen im Lungenkrebsscreening Kosteneffektivität demonstriert wurde [17, 18], sollten für sämtliche KI-Softwarelösungen, die den Anforderungen der Lungenkrebs-Früherkennungs-Verordnung entsprechen und in Deutschland potenziell zum Einsatz kommen könnten, Kosteneffektivitätsanalysen durchgeführt werden. Dies böte die Grundvoraussetzung für eine fundierte ökonomische Entscheidungsfindung hinsichtlich der Auswahl geeigneter KI-Tools und sollte von erst- sowie zweitbefundenden Zentren und sonstigen Kostenträgern unbedingt eingefordert werden.

## Herausforderung für die Integration von KI-Tools in der Radiologie

### Datenqualität und -zugänglichkeit

Eine der grundlegendsten Anforderungen für effektive KI-Systeme ist der Zugang zu großen Mengen qualitativ hochwertiger und in optimaler Weise annotierter Bilddaten. In der Praxis sind solche Datensätze jedoch oftmals unzureichend oder schwer zugänglich. Die Heterogenität der Daten, bedingt durch unterschiedliche Bildgebungsprotokolle und Geräte, erschwert zusätzlich die Entwicklung generalisierbarer KI-Modelle. Darüber hinaus bestehen Bedenken hinsichtlich des Datenschutzes in der Nutzung von Patientendaten. Zur Verbesserung der Modelle sollten Möglichkeiten geschaffen werden, die KI-Systeme an größeren Datensätzen zu trainieren.

### Integration in klinische Workflows

Die erfolgreiche Implementierung von KI erfordert deren nahtlose Einbindung in bestehende klinische Arbeitsabläufe und IT-Infrastrukturen. Allerdings besteht aktuell eine extrem hohe Zahl an unterschiedlichsten klinischen Systemen wie RIS/KIS, PACS etc., die individuelle Schnittstellenproblematiken mit sich bringen. Hier fehlt es aktuell noch an standardisierten Protokollen, welche eine reibungslose Interoperabilität zwischen KI-Systemen und bestehenden RIS gewährleisten. Zusätzlich erschwert die Vielzahl der unterschiedlichen KI-Anwendungen allein für das Lungenkrebsscreening den Aufwand der Implementierung in die Routinearbeit [19, 20].

### Validierung und Generalisierbarkeit

Die klinische Einführung von KI-Tools setzt deren umfassende Validierung voraus. Viele kommerziell verfügbaren KI-Systeme weisen jedoch eine begrenzte wissenschaftliche Evidenz auf, insbesondere hinsichtlich unabhängiger Validierungsstudien. Die Generalisierbarkeit von KI-Modellen, also deren Fähigkeit, in unterschiedlichen klinischen Kontexten und Populationen zuverlässig zu funktionieren, bleibt somit unklar. Dies erfordert umfangreiche, multizentrische Studien, die jedoch mit hohen Kosten und organisatorischem Aufwand verbunden sind [19, 20].

### Akzeptanz bei Fachpersonal

Die Einführung von KI in die Radiologie kann bei medizinischem Fachpersonal auf Skepsis stoßen. Bedenken hinsichtlich der Zuverlässigkeit von KI-Systemen, der möglichen Verdrängung menschlicher Arbeitskraft und der ethischen Implikationen können die Akzeptanz hemmen. Es ist daher essenziell, Radiolog:innen und technisches Personal frühzeitig in den Implementierungsprozess einzubinden, transparente Informationen bereitzustellen und durch gezielte Schulungen die Kompetenzen im Umgang mit KI zu fördern.

### Datenschutz und ethische Überlegungen

Der Einsatz von KI in der Radiologie wirft bedeutende Fragen des Datenschutzes und der Ethik auf. Die Verarbeitung großer Mengen sensibler Patientendaten erfordert strikte Maßnahmen zum Schutz vor unbefugtem Zugriff und Missbrauch. Zudem müssen ethische Richtlinien entwickelt werden, die den verantwortungsvollen Einsatz von KI sicherstellen, insbesondere in Bezug auf Entscheidungsprozesse, bei denen menschliches Leben betroffen ist. Da in vielen KI-Systemen die Anwendung in Cloudsystemen stattfindet, stellt sich überdies die Frage nach der Sicherheit der Daten gegenüber unbefugten Dritten [21].

### Rechtliche Aspekte

Unklar ist momentan auch die rechtliche Dimension der KI. Es stellt sich – ähnlich wie bei der Frage des autonomen Fahrens – die Frage der Haftung bei Fehlbefunden. Letztlich bleibt aktuell der Mensch in der Endverantwortung. Der Gesetzgeber muss Rahmenbedingungen schaffen, die ein sicheres Arbeiten mit KI ermöglichen [22].

## Fazit

Der Einsatz von KI im Rahmen des Lungenkrebsscreenings bietet erhebliche Chancen für eine effizientere, standardisierte und potenziell genauere Diagnostik. So zeigen Lung-CAD-Softwares vielversprechende Ergebnisse für die Detektion, Klassifikation und Verlaufsbeurteilung pulmonaler Rundherde – mit dem Potenzial, die Rolle von Radiolog:innen als Zweitbefundende zu ersetzen. Die zusätzliche Möglichkeit zur Quantifizierung von Begleitparametern wie Koronarkalk und Lungenemphysem erweitert den klinischen Nutzen solcher Systeme über die reine Krebsfrüherkennung hinaus.

Gleichzeitig bestehen technische, organisatorische, wirtschaftliche und ethisch-rechtliche Herausforderungen, die eine differenzierte Betrachtung und sorgfältige Implementierung erforderlich machen. Eine erfolgreiche Integration von KI für das Lungenkrebsscreening erfordert robuste Validierungsdaten, Kosteneffektivitätsanalysen sowie die Akzeptanz und Schulung des medizinischen Fachpersonals.

Langfristig kann KI – richtig eingesetzt – nicht nur zur Verbesserung diagnostischer Qualität, sondern auch zur Entlastung der Radiologie beitragen und Raum für eine stärkere Fokussierung auf die ärztliche Kommunikation mit den Patient:innen schaffen.
